# Effects of Melatonin Administration on Post-Stroke Delirium in Patients with Intracerebral Hemorrhage

**DOI:** 10.3390/jcm12051937

**Published:** 2023-03-01

**Authors:** Vasileios Siokas, Sara Roesch, Maria-Ioanna Stefanou, Rebecca Buesink, Vera Wilke, Jennifer Sartor-Pfeiffer, Kamaldeen Adeyemi, Sven Poli, Efthimios Dardiotis, Ulf Ziemann, Katharina Feil, Annerose Mengel

**Affiliations:** 1 Department of Neurology & Stroke, Eberhard-Karls University of Tübingen, 72076 Tuebingen, Germany; 2 Hertie Institute for Clinical Brain Research, Eberhard-Karls University of Tuebingen, 72076 Tuebingen, Germany; 3 Department of Neurology, University Hospital of Larissa, Faculty of Medicine, School of Health Sciences, University of Thessaly, 41100 Larissa, Greece

**Keywords:** intracerebral hemorrhage, post-stroke delirium, melatonin

## Abstract

Post-stroke delirium (PSD) after intracerebral hemorrhage (ICH) is considered to be even more detrimental compared to that after ischemic stroke. Treatment options for post-ICH PSD remain limited. This study aimed at investigating to what extent prophylactic melatonin administration may have beneficial effects on post-ICH PSD. We performed a mono-centric, non-randomized, non-blinded, prospective cohort study, including 339 consecutive ICH patients admitted to the Stroke Unit (SU) from December 2015 to December 2020. The cohort consisted of ICH patients who underwent standard care (defined as the control group) and ICH patients who additionally received prophylactic melatonin (2 mg per day, at night) within 24 h of ICH onset until the discharge from the SU. The primary endpoint was post-ICH PSD prevalence. The secondary endpoints were: (i) PSD duration and (ii) the duration of SU stay. The PSD prevalence was higher in the melatonin treated cohort compared to the propensity score-matched (PSM) control group. Post-ICH PSD patients receiving melatonin had shorter SU-stay durations, and shorter PSD durations, although not statistically significant. This study shows no efficacy in limiting post-ICH PSD with preventive melatonin administration.

## 1. Introduction

Post-stroke delirium (PSD) represents a severe complication of intracerebral hemorrhage (ICH) [1,2,3]. The prevalence of PSD is higher in patients with ICH compared to those with acute ischemic stroke (AIS) [4,5], with a reported frequency of 59% [2,6,7,8,9]. Post-ICH PSD develops 3 to 5 days later compared to post-AIS PSD [2,10,11]. Post-ICH PSD has been associated with increased in-hospital and 5-year mortality, prolonged intensive care unit (ICU) stay, worse functional and neurological outcomes, and worse quality of life [2,6,7].

The pathogenetic processes behind the development of post-ICH PSD are poorly understood. Defective neuronal connectivity and neurotransmission, impaired brain metabolism, neuroinflammation, disturbed sleep–wake cycles, abnormal oxygen supply, electrolytic disturbances, overt stress responses, altered cellular signaling, and brain vascular dysfunction are considered to be implicated in PSD development [12,13,14]. Despite the fact that several predisposing factors have been incriminated in increasing the risk of post-ICH PSD, the extent of their contribution to PSD has not yet been fully elucidated [2,6,15]. 

Melatonin is a hormone secreted by the pineal gland, and in clinical practice exogenous melatonin supplementation is used mainly for, but not limited to, treating sleep disorders [16]. The hypothesis of a pathophysiological implication of melatonin in delirium derives from its connection to disturbed sleep–wake systems [17], the disruption of which is a core characteristic of delirium [18]. Moreover, there is accumulating evidence in the literature for differences in melatonin levels in patients with delirium compared to healthy subjects [19,20,21]. 

With respect to the therapeutic efficacy of melatonin, conflicting data have been reported. Previous studies have indicated that melatonin supplementation may be effective in preventing delirium, however, there are also studies reporting no benefit thereof [22,23]. Exogenous melatonin was associated with a significant attenuation of PSD risk in AIS, and consequently with improved clinical outcomes in PSD stroke patients [24]. While there are no large studies today on the effects of melatonin on post-ICH PSD, there is some evidence that melatonin supplementation may improve clinical outcomes (duration of mechanical ventilation, length of ICU stay, clinical manifestations) in ICH patients [25,26].

In view of the former considerations, we hypothesized that preventive administration of melatonin will decrease the frequency of post-ICH PSD and also demonstrate beneficial effects on related outcomes (e.g., PSD duration and SU-stay duration). As such, the aim of the present study in a cohort of ICH patients was to examine the effect of preventive administration of melatonin on post-ICH PSD prevalence. We also examined the effect of preventive administration of melatonin on PSD duration and SU-stay duration.

## 2. Materials and Methods

### 2.1. Study Design and Regulations

We conducted a mono-centric, non-randomized, non-blinded, prospective cohort study. Our cohort consisted of ICH patients who were prophylactically treated with melatonin for PSD prevention versus non-treated ICH patients (defined as the control group). The institutional ethics committee approved the protocol of the study (protocol number 752/2018BO2). Due to the clinic-wide consent regarding the use of de-identified routine treatment data for research purposes, the individual informed consent from the participants was waived.

### 2.2. Study Population

Consecutive ICH patients admitted to the stroke unit (SU) of the University Hospital of Tübingen, between 1 December 2015 and 31 December 2020 were included. All patients were diagnosed with nontraumatic ICH according to the International Classification of Diseases, 10th Revision I61.

### 2.3. Participants’ Exclusion Criteria

The following exclusion criteria were applied: (1) duration of SU stay <24 h; (2) diagnosis of delirium caused by benzodiazepines on admission (oa); (3) a Richmond Agitation-Sedation Scale (RASS) level of −5 or −4 for the majority (>50%) of the stay; (4) patients on mechanical ventilation or in shock or who had severe liver or renal insufficiency; (5) an incomplete record of the RASS and Intensive Care Delirium Screening Checklist (ICDSC) during the stay; (6) patients who underwent neurosurgical intervention immediately after baseline-CT; (7) patients with traumatic ICH.

### 2.4. Melatonin Administration

All treated patients underwent prophylactic melatonin supplementation according to the standard operating procedure (SOP) of the SU for PSD prevention, which has previously been described in detail [24].

In brief, the rationale for preventive application of melatonin in the SOP were: (i) the lack of clear evidence regarding therapeutic options for PSD prevention and management; (ii) melatonin’s excellent tolerability and safety profile; and (iii) evidence regarding the beneficial effect of melatonin in delirium prevention in elderly, in stroke patients, and in ICU patients [24,25,26,27]. The treated cohort received melatonin orally (and in case of severe dysphagia administered through a nasogastric tube) within the first 24 h of ICH onset (single dose of 2 mg/day at 8 p.m.) [28] until discharge from the SU.

### 2.5. Data Collection

The baseline characteristics, personal medical history, and in-hospital clinical parameters for all the participants were retrieved from the clinical information system (Intellispace Critical Care and Anesthesia information system; Philips Healthcare). Data from head neuroimaging (CT or MRI) on admission were also obtained. 

We applied the ICDSC in order to assess PSD in all participants (both melatonin-treated and control cohort) [1]. The ICDSC scores were recorded on admission and every 8 h (morning, afternoon, and night) during the whole SU stay, from trained neurocritical care nurses. Participants were considered to have PSD in case of ISDSC ≥4 for non-aphasic and ≥5 for aphasic patients [1,29], while the first pathological score was indicative of the PSD onset. The diagnosis of PSD was made based on the Diagnostic and Statistical Manual of Mental Disorders (DSM)-5 criteria, by neurologists blinded to the ICDSC scores [24]. The validity and reliability of ICDSC in measuring delirium in stroke patients have been assessed in a previous study [1]. Data regarding PSD duration during the SU stay were also gathered.

The following data were collected for each participant: age, sex, history of hypertension (HP), history of diabetes mellitus (DM), history of hypercholesterolemia (HCL), history of atrial fibrillation (AF), history of coronary artery disease (CAD), obesity (defined as body mass index [BMI] > 30), chronic renal failure, chronic hepatic failure, history of smoking, history of alcohol consumption, history of malignancy, history of depression, cognitive status (normal cognition vs. mild cognitive impairment (MCI/dementia)), infections during stay at SU, NIHSS oa, presence of aphasia oa, ICH-score oa, modified Rankin scale (mRS) before the index event, the etiology of ICH (hypertension, cerebral amyloid angiopathy (CAA), mass (benign and non-benign), oral anticoagulant (OAC), vessel pathology, other/unknown), hemorrhagic location at baseline (deep white matter (DWM), lobar, brainstem, cerebellum, left or right hemisphere), intraventricular hemorrhage (IVH) oa, IVH extension, ICH volume (expressed as cm^3^), invasive procedures (surgical evacuation and external ventricular drain [30]), and SU-stay duration.

### 2.6. Study Endpoints

The primary endpoint of the current study was the effect of preventive melatonin application on post-ICH PSD prevalence. The secondary endpoints were the effects of melatonin administration on PSD duration and SU-stay duration.

### 2.7. Statistical Analysis

To minimize selection bias of the retrospective analysis, propensity score matching (PSM) with a match tolerance of 0.2 was performed in order to balance baseline differences in clinical covariates between patients treated with melatonin and controls. Standardized mean differences (SMD) were calculated to compare participant features after PSM. SMD values greater than 0.25 were considered indicative for imbalance [31,32].

We used the Shapiro–Wilk test to check the distribution of the data. For categorical variables, the differences between groups regarding demographics and clinical characteristics were assessed using Pearson’s chi-squared test. For continuous variables, we applied Mann–Whitney U tests due to non-normal distribution of the data. Values are expressed as total number (n) with the respective percentage (%) for categorical variables. For continuous variables, values are expressed as medians with the respective interquartile ranges (IQR).

We used chi-square tests to examine the difference in post-ICH PSD prevalence, between the melatonin-treated cohort and the control group. To better capture the treatment effect on each group (for descriptive purposes) between group differences regarding PSD duration and SU-stay duration, we applied Mann–Whitney U tests. For the effect of the preventive administration of melatonin on the PSD duration (hours) and SU-stay duration (hours), we applied linear regression analyses, using melatonin treatment as predictor, after logarithmic transformation of the dependent variables (i.e., PSD duration and SU-stay duration) due to non-normal distribution of the data. Adjusted models for the variables that differed between groups or correlated with the outcome of interest in univariate analysis, were also performed. The multicollinearity was assessed by using the variance inflation factor (VIF). VIF values of 10 were considered as cut points for the elimination variable from the model [33]. No multicollinearity was detected (VIF < 10) in any of the analyses.

For the statistically significant results, we also performed sensitivity analyses by excluding patients that died during hospitalization, aiming to eliminate any possible latent effect of this factor on the PSD duration or SU-stay duration. 

An alpha error of 5% (*p* < 0.05) was set as the level of statistical significance for all procedures. All statistical analyses were performed with SPSS (Version 29, IBM, Armonk, NY, USA).

## 3. Results

### 3.1. Participants’ Characteristics and Post-ICH PSD Prevalence

In total, 339 patients with ICH were included in this study. Out of the 339 ICH patients, 127 (37.5%) developed PSD during the SU stay. The majority of them (74.2%) developed PSD within the first 48 h of admission, whereas the vast majority of them (∼90%) developed PSD within the first 120 h after admission (Figure S1). Median of PSD onset was 27.5 h (IQR, 44), and its median duration was 144 h (IQR, 157). The post-ICH PSD cohort was older and had higher NIHSS and ICH-scores oa, compared to their non-PSD counterparts. Moreover, we found an increased prevalence of AF, infections during SU stay, IVH extension, hypertension, and OAC as etiology of ICH in the post-ICH PSD cohort. The vessel pathology as etiology for ICH was more prevalent in non-PSD patients. Patients’ characteristics are presented in Table 1.

### 3.2. Effect of Melatonin Administration on PSD Prevention

The melatonin-treated cohort had lower NIHSS, ICH-scores, and ICH-volume oa, increased prevalence of HCL, OAC as etiology of ICH, and decreased prevalence of EVD, surgical evacuation, and IVH extension compared to the control group. After the PSM, no statistically significant between-group differences in characteristics were noted, with the exception of brainstem location of ICH at baseline, which was higher in the control group. Patients’ characteristics, based on receiving (or not) melatonin, are presented in Table 2.

The PSD prevalence was higher in the melatonin-treated cohort (47.9%) compared to the control group (31.8%, *p* = 0.004), and compared to the PSM control group (26.3%, *p* < 0.001).

#### 3.2.1. Effect of Melatonin Administration on PSD Duration

Post-ICH PSD patients’ characteristics, based on receiving (or not) melatonin after the PSM in the initial sample (n = 88) are presented in Table 3. No statistically significant between-group differences in characteristics were noted, with the exception of NIHSS oa, which was statistically significant higher in the control group.

**Figure 1:**
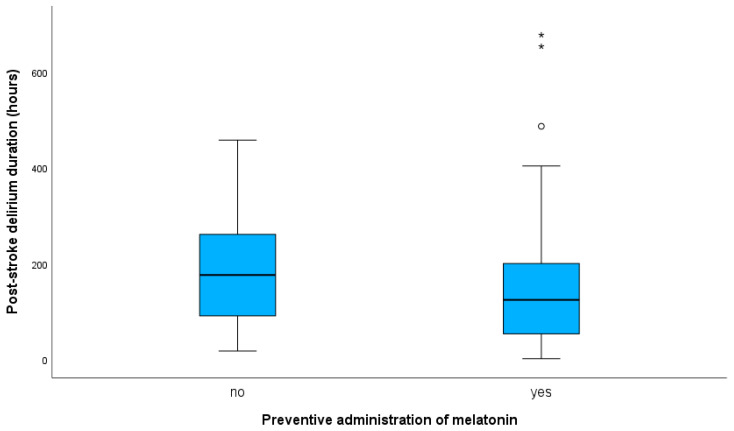
Box plots presenting data for post-stroke delirium duration (hours) in post-intracerebral hemorrhage patients with respect to administration (or not) of melatonin. In the box plot, the black line within a box indicates the median, the boundary of the box closest to zero indicates the 1st quartile, and the boundary of the box farthest from zero indicates the 3rd quartile. Outliers with values more than 3 interquartile ranges (IQRs) from the end of the box are denoted with an asterisk (*). Outliers with values more than 1.5 IQRs, but less than 3 IQRs from the end of the box, are denoted with a circle (o). Whiskers above and below the box indicate max and min values, respectively, not including outliers. Median duration of delirium in patients receiving melatonin was 124 h, and 176 h in the control group.

In PSD patients, preventive melatonin administration did not correlate with duration of PSD in neither crude (b, −0.189; 95% CI, −0.407 to 0.029; *p* = 0.088) nor adjusted models (b, −0.071; 95% CI, −0.273 to 0.131; *p* = 0.436). Adjustment was made for variables that significantly correlated with PSD duration, as presented at Table S1, or differed between treated and control PSD group (Table 3). Results regarding the effect of preventive administration of melatonin on PSD duration (unadjusted and adjusted analyses) are summarized in Table 4. Median duration of delirium in PSD patients receiving melatonin was 124 h (IQR, 151), and 176 h (IQR, 186) in the control group (*p*-value for Mann–Whitney U test = 0.104). Box plots presenting data for PSD duration (hours) with respect to administration (or not) of melatonin are shown in Figure 1. 

#### 3.2.2. Effect of Melatonin Administration on SU-Stay Duration

##### Post-ICH PSD Patients

**Figure 2:**
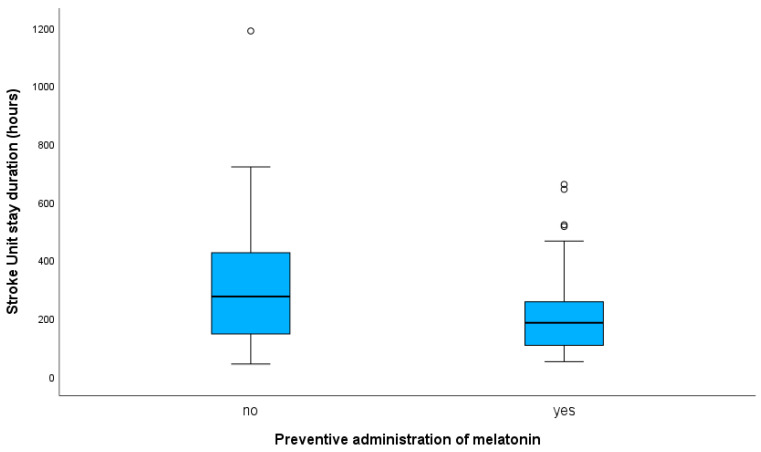
Box plots presenting data for stroke-unit-stay duration (hours) in respect to administration (or not) of melatonin in post-intracerebral hemorrhage patients with post-stroke delirium. In the box plot, the black line within a box indicates the median, the boundary of the box closest to zero indicates the 1st quartile, and the boundary of the box farthest from zero indicates the 3rd quartile. Outliers with values more than 1.5 IQRs, but less than 3 IQRs from the end of the box, are denoted with a circle (o). Whiskers above and below the box indicate max and min values, respectively, not including outliers. Median stroke-unit-stay duration of post-stroke delirium patients receiving melatonin was 184 h, and 274 h in the control group (*p*-value for Mann–Whitney U test = 0.033).

Melatonin administration was negatively correlated with SU-stay duration of PSD patients (b, −0.147; 95% CI, −0.278 to −0.016; *p* = 0.028). The significance did not remain in the adjusted model (b, −0.094; 95% CI, −0.207 to 0.020; *p* = 0.104). Adjustment was made for variables that significantly correlated with SU-stay duration, as presented in Table S2 or differing between treated and control PSM PSD groups (Table 3). Median SU-stay duration of PSD patients receiving melatonin was 184 h (IQR, 154), and 274 h (IQR, 285) in the PSD control group (*p*-value for Mann–Whitney U test = 0.033). Box plots presenting data for SU-stay duration (hours) in respect to administration (or not) of melatonin in PSD are shown in Figure 2. 

However, the significance did not remain in the sensitivity analysis, after excluding patients who died during hospitalization (*p*-value for Mann–Whitney U test = 0.051), although post-ICH PSD patients receiving melatonin had lower SU-stay duration compared to the control group (187 h vs. 274 h) (Figure S2). 

##### Post-ICH without PSD Patients

Post-ICH without PSD patients’ characteristics, based on receiving (or not) melatonin after the PSM in the initial sample (n = 149), are presented in Table S3. Melatonin administration was not associated with SU-stay duration of non-PSD patients in both crude (b, −0.004; 95% CI, −0.114 to 0.123; *p* = 0.941) and adjusted (b, 0.031; 95% CI, −0.60 to 0.131; *p* = 0.460) models. Adjustment was made for variables that significantly correlated with SU-stay duration, as presented in Table S2, or differed between treated and control PSM non-PSD groups (Table S3). Median SU-stay duration of non-PSD patients receiving melatonin was 108 h (IQR, 161), and 109 h (IQR, 154) in the non-PSD control group (*p*-value for Mann–Whitney U test = 0.910). Box plots presenting data for SU-stay duration (hours) in respect to administration (or not) of melatonin in non-PSD are shown in Figure S3. 

Results regarding the effect of preventive administration of melatonin on SU-stay duration (unadjusted and adjusted analyses) are summarized in Table 5.

## 4. Discussion

In this cohort study, we investigated the potential preventive effect of low-dose melatonin on PSD. The PSD prevalence was higher in the melatonin cohort compared to control group. Since age of treated patients was slightly higher compared to controls after PSM (76 vs. 74.5 years), it may comprise a possible reason for these results. Moreover, the lack of a preventive effect of melatonin on PSD prevalence may be attributed to the low melatonin dosage in our study, especially when it is compared to melatonin dosages of previous studies reporting a beneficial effect of melatonin on ICH [25,34].

The preventive administration of melatonin had marginal beneficial effects on SU-stay duration and on PSD duration in post-ICH patients. More precisely, the post-ICH patients receiving melatonin appeared to have shorter, but not statistically significant, PSD duration compared to untreated patients. Of note, in our study, median SU-stay duration of PSD patients receiving melatonin was shorter compared to the PSD control group, highlighting a possible beneficial effect of the preventive melatonin administration in ICH patients. In particular, the median SU-stay duration of PSD patients receiving melatonin was reduced by more than 3.5 days compared to their non-treated PSD counterparts. Considering this, we could hypothesize that melatonin may have a beneficial effect only on specific PSD parameters, while higher melatonin dosages may be required for preventive effects on PSD prevalence to become evident. 

The vast majority of post-ICH PSD patients (~90%) developed PSD within the first 120 h after admission. By contrast, one previous report [24] suggests that post-AIS PSD manifests earlier compared to post-ICH PSD. The vast majority of AIS patients (96.7%) developed PSD within the first 72 h [24]. A possible explanation for these findings could be that secondary ischemic/hypoxic dysfunction in tissue surrounding the ICH, may lead to a delayed PSD occurrence in ICH compared to AIS, albeit the exact pathophysiological mechanisms remain to be elucidated [35,36]. Delayed post-ICH PSD development and overlapping clinical features with ICH [2] suggest that physicians should be alerted to detect PSD in ICH patients during almost their entire stay in SU. 

Among the strengths of our study is the inclusion of a large number of well-characterized ICH patients, following a standardized protocol for PSD detection. Secondly, we performed multivariate analyses with adjustment for a large amount of potential cofounding factors, sensitivity analyses for the statistically significant results, and mainly a PSM analysis in an attempt to minimize any possible selection bias. At this point we must also acknowledge the limitations of our study. Firstly, we did not randomize the sample for melatonin administration and the generalizability of our results is limited, despite our approach of using PSM analysis. Thus, the present findings require validation from a prospective randomized controlled trial (RCT). Moreover, given the different pathophysiological mechanisms implicated in different PSD subtypes [37], additional studies in subclusters of patients based on PSD subtype, will allow us to draw more robust conclusions regarding the effect of melatonin on post-ICH PSD. 

## 5. Conclusions

This study shows no efficacy in limiting post-ICH PSD with preventive melatonin administration, as the PSD prevalence was higher in the melatonin cohort. However, the preventive administration of melatonin appears to have marginal beneficial effects on SU-stay duration and on PSD duration in post-ICH PSD. Yet, due to methodological limitations related to the non-randomized study design, we cannot totally exclude that the utility of melatonin may have been underestimated. Taking into consideration the limited available therapeutic strategies for PSD, large-scale RCTs are warranted to further test and validate the role of melatonin in preventing and treating post-ICH PSD.

## Figures and Tables

**Table 1:** Characteristics of patients with ICH.

Patients’ Characteristics	All ICH Patients n = 339	ICH Patients with PSD n = 127	ICH Patients without PSD n = 212	*p*-Value
**Demographics**				
Age, y median, (IQR)	74.00 (20.00)	78.00 (16.00)	71.50 (22.00)	**0.001 ^**
Sex, n (%)				0.454 *
Male	186 (54.9)	73 (57.5)	113 (53.3)	
Female	153 (45.1)	54 (42.5)	99 (46.7)	
**Risk Factors,** n (%)				
HP	268 (79.1)	104 (81.9)	164 (77.4)	0.321 *
DM	64 (18.9)	27 (21.3)	37 (17.5)	0.386 *
HCL	67 (19.8)	28 (22.0)	39 (18.4)	0.414 *
AF	94 (27.7)	46 (36.2)	48 (22.6)	0.007 *
CAD	60 (17.8)	26 (20.5)	34 (16.1)	0.310 *
Obesity (BMI > 30)	61 (18.0)	22 (17.3)	39 (18.4)	0.803 *
Chronic renal failure	38 (11.2)	15 (11.8)	23 (10.8)	0.786 *
Chronic hepatic failure	8 (2.4)	2 (1.6)	6 (2.8)	0.461 *
Smoking	46 (13.6)	15 (11.8)	31 (14.6)	0.464 *
Alcohol	27 (8.0)	10 (7.9)	17 (8.0)	0.962 *
Malignancy	36 (10.2)	12 (9.4)	24 (11.3)	0.588 *
Depression	21 (6.2)	7 (5.5)	14 (6.6)	0.686 *
Cognition				0.153 *^#^
Health	301 (89.6)	109 (86.5)	192 (91.4)	
MCI	31 (9.2)	13 (10.3)	18 (8.6)	
Dementia	4 (1.2)	4 (3.2)	0 (0.0)	
Any kind of infection	176 (51.9)	85 (66.9)	91 (42.9)	<0.001 *
**Baseline clinical variables/scales**				
NIHSS oa, median (IQR)	7.00 (10.0)	8.00 (9.00)	6.00 (11.00)	**0.005 ^**
Aphasia oa, n (%)	125 (37.4)	53 (41.7)	72 (34.8)	0.203 *
ICH-score oa, median (IQR)	1.00 (1.00)	2.00 (1.00)	1.00 (2.00)	**<0.001 ^**
mRS before the ICH, median (IQR)	0.00 (2.00)	1.00 (2.00)	0.00 (1.00)	0.131 ^
**Etiology,** n (%)				
Hypertension	191 (56.3)	82 (64.6)	109 (51.4)	0.018 *
CAA	27 (8.0)	12 (9.4)	15 (7.1)	0.435 *
Mass	26 (7.7)	8 (6.3)	18 (8.5)	0.463
OAC	92 (27.2)	44 (34.6)	48 (22.6)	0.016 *
Vessel pathology	20 (5.9)	2 (1.6)	18 (8.5)	0.009 *
Other/unknown	67 (19.8)	21 (16.5)	46 (21.7)	0.248 *
**Location at baseline,** n (%)				
DWM	157 (46.6)	65 (51.2)	92 (43.4)	0.189 *
Lobar	139 (41.0)	46 (36.2)	93 (43.9)	0.166 *
Brainstem	18 (5.3)	4 (3.2)	14 (6.6)	0.172 *
Cerebellum	41 (12.2)	15 (11.8)	26 (12.4)	0.877 *
Left hemisphere	182 (53.7)	65 (51.2)	117 (55.2)	0.474 *
Right hemisphere	174 (51.3)	64 (50.4)	110 (51.9)	0.790 *
IVH oa	5 (1.5)	2 (1.6)	3 (1.4)	0.906 *
IVH extension	139 (41.2)	66 (52.4)	73 (34.6)	0.001 *
**ICH Volume (cm^3^),** median (IQR)	11.00 (22.00)	11.50 (23.00)	10.50 (22.00)	0.478 ^
**Invasive procedures** n (%)				
Surgical evacuation	49 (14.5)	13 (10.2)	36 (17.0)	0.087 *
EVD	49 (14.5)	24 (18.9)	25 (11.8)	0.078 *

ICH, intracerebral hemorrhage; PSD, post-stroke delirium; HP; hypertension; DM, diabetes mellitus; HCL, hypercholesterolemia; AF, atrial fibrillation; CAD, coronary artery disease; BMI, body mass index; MCI, mild cognitive impairment; NIHSS, National Institutes of Health Stroke Scale; mRS, modified Rankin scale; CAA, cerebral amyloid angiopathy; OAC, oral anticoagulants; DWM, deep white matter; IVH, intraventricular; oa, on admission; EVD, external ventricular drain. Valid percent: ^ Mann–Whitney U test, * chi-square test, ^#^ MCI/dementia vs. healthy cognition. Statistically significant values are given in bold.

**Table 2:** Characteristics of patients treated with melatonin vs. the control cohort.

Patients’ Characteristics	Melatonin Treated n = 119	Control Cohort n = 220	*p*-Value ^1^	PSM Control Cohort n = 118	*p*-Value ^2^	SMD ^2^
**Demographics**						
Age, y median, (IQR)	76.00 (18.00)	74.00 (21.00)	0.059	74.50 (22.00)	0.136 ^	0.014
Sex, n (%)			0.536 *		0.597 *	0.053
Male	68 (57.1)	118 (53.6)		71 (60.2)		
Female	51 (42.9)	102 (46.4)		47 (39.8)		
**Risk Factors,** n (%)						
HP	101 (84.9)	167 (75.9)	0.053 *	93 (78.8)	0.238 *	0.159
DM	19 (16.0)	45 (20.5)	0.314 *	27 (22.9)	0.189 *	0.175
HCL	34 (28.1)	33 (15.0)	0.003 *	24 (20.3)	0.131 *	0.116
AF	32 (26.9)	62 (28.2)	0.800 *	38 (32.2)	0.393 *	0.183
CAD	26 (21.8)	34 (15.5)	0.146 *	22 (18.8)	0.539 *	0.075
Obesity (BMI > 30)	20 (16.8)	41 (18.6)	0.676 *	24 (20.3)	0.504 *	0.090
Chronic renal failure	15 (12.6)	23 (10.5)	0.549 *	14 (11.9)	0.843 *	0.021
Chronic hepatic failure	2 (1.7)	6 (2.7)	0.545 *	3 (2.5)	0.651 *	0.056
Smoking	14 (11.8)	32 (14.5)	0.475 *	23 (19.5)	0.107 *	0.213
Alcohol	10 (8.4)	17 (7.5)	0.826 *	10 (8.5)	1.000 *	0.004
Malignancy	13 (10.9)	23 (10.5)	0.893 *	15 (12.7)	0.687 *	0.056
Depression	6 (5.0)	15 (6.8)	0.517 *	7 (5.9)	0.775 *	0.040
Cognition			0.822 *^#^		0.393 *^#^	0.107
Health	106 (89.1)	195 (89.9)		107 (92.2)		
MCI	11 (9.2)	20 (9.2)		9 (7.8)		
Dementia	2 (1.7)	2 (0.9)		0 (0.0)		
Any kind of infection	55 (46.2)	121 (55.0)	0.122 *	61 (51.7)	0.436 *	0.110
**Baseline clinical variables/scales**						
NIHSS oa, median (IQR)	5.00 (8.0)	9.00 (11.00)	**<0.001 ^**	6.00 (9.00)	0.076 **^**	0.029
Aphasia oa, n (%)	38 (31.9)	87 (40.5)	0.123 *	42 (35.6)	0.582 *	0.078
ICH-score oa, median (IQR)	1.00 (2.00)	2.00 (1.00)	**0.003 ^**	1.00 (1.00)	0.097 **^**	0.213
mRS before the ICH, median (IQR)	0.00 (1.00)	1.00 (2.00)	0.128 ^	0.00 (2.00)	0.524 ^	0.000
**Etiology**						
Hypertension	69 (58.0)	122 (55.5)	0.645 *	66 (55.9)	0.693 *	0.042
CAA	12 (10.1)	15 (6.8)	0.289 *	7 (5.9)	0.232 *	0.155
Mass	9 (7.6)	17 (7.9)	0.957 *	9 (7.6)	1.000 *	0.000
OAC	42 (35.3)	50 (22.7)	0.013 *	32 (27.1)	0.161 *	0.178
Vessel pathology	4 (3.4)	16 (7.3)	0.145 *	10 (8.5)	0.098 *	0.217
Other/unknown	25 (21.0)	42 (19.1)	0.672 *	17 (14.4)	0.229 *	0.174
**Location at baseline**						
DWM	53 (45.3)	104 (47.3)	0.730 *	57 (48.3)	0.689 *	0.060
Lobar	47 (39.5)	92 (41.8)	0.678 *	43 (36.4)	0.687 *	0.064
Brainstem	3 (2.5)	15 (6.8)	0.093 *	10 (8.5)	0.046 *	0.265
Cerebellum	15 (12.6)	26 (11.9)	0.856 *	16 (13.7)	0.827 *	0.033
Left hemisphere	68 (57.1)	114 (51.8)	0.348 *	70 (59.3)	0.792 *	0.045
Right hemisphere	56 (47.1)	118 (53.6)	0.247 *	56 (47.5)	0.896 *	0.008
IVH oa	1 (0.8)	4 (1.8)	0.476 *	3 (2.5)	0.313 *	0.134
IVH extension	40 (33.6)	99 (45.4)	0.035 *	48 (40.7)	0.282 *	0.147
**ICH Volume (cm^3^)**	8.00 (12.00)	13 (27.00)	**0.004 ^**	7.00 (22.00)	0.997 **^**	0.009
**Invasive procedures,** n (%)						
Surgical evacuation	8 (6.7)	41 (18.6)	0.003 *	13 (11.0)	0.161 *	0.152
EVD	9 (7.6)	40 (18.3)	0.007 *	14 (11.9)	0.272 *	0.145

ICH, intracerebral hemorrhage; PSD, post-stroke delirium; PSM, propensity-score matched; SMD, standardized mean difference (absolute values); HP, hypertension; DM, diabetes mellitus; HCL, hypercholesterolemia; AF, atrial fibrillation; CAD, coronary artery disease; BMI, body mass index; MCI, mild cognitive impairment; NIHSS, National Institutes of Health Stroke Scale; oa, on admission; mRS, modified Rankin scale; CAA, cerebral amyloid angiopathy; OAC, oral anticoagulants; DWM, deep white matter; IVH, intraventricular; EVD, external ventricular drain. Valid percent: ^ Mann–Whitney U test, * chi-square test, ^#^ MCI/dementia vs. healthy cognition. ^1^ Comparison between melatonin-treated cohort and control group. ^2^ Comparison between melatonin-treated cohort and PSM control group. Statistically significant values are given in bold.

**Table 3:** Characteristics of patients with post-ICH PSD treated with melatonin vs. the control cohort after propensity matching.

Patients’ Characteristics	PSD Melatonin Treated n = 57	PSD Control Cohort n = 31	*p*-Value
**Demographics**			
Age, y median, (IQR)	80.00 (16.00)	79.00 (16.00)	0.624 ^
Sex, n (%)			0.640 *
Male	32 (56.1)	19 (61.3)	
Female	25 (43.9)	12 (38.7)	
**Risk Factors,** n (%)			
HP	51 (89.5)	25 (80.6)	0.249 *
DM	12 (21.1)	8 (25.8)	0.611 *
HCL	19 (33.3)	6 (19.4)	0.165 *
AF	19 (33.3)	14 (45.2)	0.274 *
CAD	15 (26.3)	9 (29.0)	0.785 *
Obesity (BMI > 30)	10 (17.5)	6 (19.4)	0.833 *
Chronic renal failure	8 (14.0)	4 (12.9)	0.883 *
Chronic hepatic failure	1 (1.8)	0 (0.0)	0.458 *
Smoking	6 (10.5)	5 (16.1)	0.448 *
Alcohol	7 (12.3)	1 (3.2)	0.158 *
Malignancy	4 (7.0)	4 (12.9)	0.359 *
Depression	2 (3.5)	3 (9.7)	0.232 *
Cognition			0.570 *^#^
Health	47 (82.5)	27 (87.1)	
MCI	8 (14.0)	4 (12.9)	
Dementia	2 (3.5)	0 (0.0)	
Any kind of infection	33 (57.9)		0.067 *
**Baseline clinical variables/scales**			
NIHSS oa, median (IQR)	6.00 (8.00)	10.00 (8.00)	**0.018 ^**
Aphasia oa, n (%)	20 (35.1)	16 (51.6)	0.132 *
ICH-score oa, median (IQR)	2.00 (1.00)	2.00 (1.00)	0.305 **^**
mRS before the ICH, median (IQR)	0.50 (3.00)	0.00 (1.00)	0.190 ^
**Etiology**			
Hypertension	39 (68.4)	18 (58.1)	0.331 *
CAA	7 (12.3)	3 (9.7)	0.713 *
Mass	2 (3.5)	4 (12.9)	0.095 *
OAC	24 (42.1)	10 (32.3)	0.365 *
Vessel pathology	0 (0.40)	0 (0.0)	NA *
Other/unknown	9 (15.8)	4 (12.9)	0.715 *
**Location at baseline**			
DWM	31 (54.4)	14 (45.2)	0.408 *
Lobar	20 (35.1)	13 (41.9)	0.526 *
Brainstem	0 (0.0)	2 (6.5)	0.054 *
Cerebellum	6 (10.5)	4 (12.9)	0.737 *
Left hemisphere	32 (56.1)	18 (58.1)	0.862 *
Right hemisphere	27 (47.4)	13 (41.9)	0.625 *
IVH oa	1 (1.8)	0 (0.0)	0.458 *
IVH extension	28 (49.1)	16 (51.6)	0.823 *
**ICH Volume (cm^3^)**	9.00 (17.00)	10.00 (23.00)	0.958 **^**
**Invasive procedures,** n (%)			
Surgical evacuation	1 (1.8)	2 (6.5)	0.246 *
EVD	5 (8.8)	5 (16.1)	0.299 *

ICH, intracerebral hemorrhage; PSD, post-stroke delirium; HP, hypertension; DM, diabetes mellitus; HCL, hypercholesterolemia; AF, atrial fibrillation; CAD, coronary artery disease; BMI, body mass index; MCI, mild cognitive impairment; NIHSS, National Institutes of Health Stroke Scale; oa, on admission; mRS, modified Rankin scale; CAA, cerebral amyloid angiopathy; OAC, oral anticoagulants; DWM, deep white matter; IVH, intraventricular; EVD, external ventricular drain. Valid percent: ^ Mann–Whitney U test, * chi-square test, ^#^ MCI/dementia vs. healthy cognition. Statistically significant values are given in bold.

**Table 4:** Effect of melatonin administration on PSD duration.

	PSD Cohort	
PSD Duration	B (95CI)	*p*-Value
Unadjusted	−0.189 (−0.407–0.029)	0.088
Adjusted ^	−0.071 (−0.273–0.131)	0.486

B, unstandardized correlation coefficient; CI, confidence interval; PSD, post-stroke delirium. ^ Adjusted for the variables associated with PSD duration in univariate binary linear regression (Table S1) or differing between treated and control PSM PSD groups (Table 3).

**Table 5:** Effect of melatonin administration on SU-stay duration.

	PSD Cohort		Non-PSD Cohort	
SU-Stay Duration	B (95CI)	*p*-Value	B (95CI)	*p*-Value
Unadjusted	−0.147 (−0.278–−0.016)	**0.028**	−0.004 (−0.114–0.123)	0.941
Adjusted *	−0.094 (−0.207–0.020)	0.104	0.036 (−0.60–0.131)	0.460

SU, stroke unit; B, unstandardized correlation coefficient; CI, confidence interval; PSD, post-stroke delirium. * The PSD group adjusted for the variables that significantly correlated with SU-stay duration (Table S2) or differed between treated and control PSM PSD groups (Table 3). The non-PSD group is adjusted for the variables that significantly correlated with SU-stay duration (Table S2) or differed between treated and control PSM non-PSD groups (Table S3). Statistically significant values are given in bold.

## Data Availability

The datasets used and analyzed during the current study are available from the corresponding author on reasonable request.

## Supplementary Materials

The following supporting information can be downloaded at: https://www.mdpi.com/article/10.3390/jcm12051937/s1, Table S1: Analysis for effect of factors on PSD duration; Table S2: Analysis for effect of factors on SU-stay duration; Table S3: Post-ICH without PSD patients’ characteristics treated with melatonin vs. the control cohort after propensity matching; Figure S1: Cumulative post-stroke delirium prevalence (line) and point prevalence (bar plot); Figure S2: Box plots presenting data for stroke unit stay duration (hours) in respect to administration (or not) of melatonin in post-intracerebral hemorrhage patients with post-stroke delirium, who did not die during hospitalization; Figure S3: Box plots presenting data for stroke unit stay duration (hours) with respect to administration (or not) of melatonin in post-intracerebral hemorrhage patients without post-stroke delirium.
