# Supplementary material for: Effects of Melatonin Administration on Post-Stroke Delirium in Patients with Intracerebral Hemorrhage
Source: J Clin Med. 2023 Mar 1;12(5):1937. doi: 10.3390/jcm12051937 (PMC10004342; doi:10.3390/jcm12051937)
Supplement: Supplementary file 1 [file jcm-12-01937-s001.zip › jcm-2213432-supplementary.pdf]

Table S1. Analysis for effect of factors on PSD duration.

	PSD cohort	
	**PSD duration**	
	B (95CI)	p-value
**Demographics**		
Age	-0.006 (-0.015- 0.003)	0.206
Sex	-0.238 (-0.448- -0.028)	**0.027**
**Risk Factors**		
HP	-0.109 (-0.208- 0.427)	0.495
DM	0.091 (-0.160- 0.342)	0.473
HCL	0.080 (-0.157- 0.316)	0.504
AF	0.166 (-0.052- 0.383)	0.134
CAD	0.058 (-0.182- 0.298)	0.634
Obesity (BMI > 30)	0.434 (0.162- 0.706)	**0.002**
Chronic renal failure	0.184 (-0.120- 0.488)	0.233
Chronic hepatic failure	0.262 (-0.727- 1.251)	0.599
Smoking	0.339 (0.015- 0.662)	**0.040**
Alcohol	0.238 (-0.147- 0.623)	0.223
Malignancy	0.329 (-0.688- 0.029)	0.071
Depression	0.142 (-0.311- 0.595)	0.534
MCI/Dementia	-0.133 (-0.428- 0.163)	0.375
Any kind of infections	0.349 (0.141- 0.558)	**0.001**
**Baseline clinical variables/scales**		
NIHSS oa	0.024 (0.007- 0.041)	**0.007**
Aphasia oa	0.073 (-0.143- 0.289)	0.505
ICH-score before the ICH	0.077 (-0.039- 0.194)	0.189
mRS oa	-0.056 (-0.135- 0.023)	0.165
**Etiology**		
Hypertension	0.226 (0.008- 0.444)	**0.043**
CAA	-0.430 (-0.764- -0.095)	**0.012**
Mass	-0.336 (-0.747- 0.074)	0.107
OAC	0.065 (-0.155- 0.285)	0.558
Vessel Pathology	NA	NA
Other/Unknown	0.189 (-0.105- 0.483)	0.204
**Location at baseline**		
DWM	0.297 (0.093- 0.501)	**0.005**
Lobar	-0.163 (-0.382- 0.056)	0.142
Brainstem	0.495 (-0.205- 1.196)	0.163
Cerebellum	-0.322 (-0.646- 0.002)	0.051
Left hemisphere	-0.046 (-0.261- 0.168)	0.668
Right hemisphere	0.031 (-0.183- 0.245)	0.773
IVH oa	NA	NA
IVH extension	0.223 (0.015- 0.431)	**0.036**
**ICH Volume [cm³]**	0.077 (-0.039- 0.194)	0.189
**Invasive procedures**		
Surgical evacuation	-0.126 (-0.704- 0.452)	0.667
EVD	0.415 (0.096- 0.734)	**0.011**

B, unstandardized correlation coefficient; OR, odds ratio; CI, confidence interval; ICH, intracerebral hemorrhage; PSD, post-stroke delirium; HP; hypertension; DM, Diabetes mellitus; HCL, Hypercholesterolemia; AF, atrial fibrillation; CAD, coronary artery disease; BMI, body mass index; MCI, mild cognitive impairment; NIHSS, National Institutes of Health Stroke Scale; oa, on admission; mRS, modified Rankin Scale; CAA, cerebral amyloid angiopathy; OAC, oral anticoagulants; DWM, deep white matter; IVH, intraventricular; EVD, external ventricular drain
Statistically significant values are given in bold

Table S2. Analysis for effect of factors on SU-stay duration.

	PSD cohort		Non-PSD cohort	
	B (95CI)	p-value	B (95CI)	p-value
**Demographics**				
Age	-0.008 (-0.013- -0.003)	**0.002**	0.0001 (-0.004- 0.004)	0.932
Sex	-0.170 (-0.295- -0.045)	**0.008**	0.103 (-0.014- 0.220)	0.085
**Risk Factors**				
HP	0.187 (0.03- 0.370)	**0.046**	0.222 (0.083- 0.361)	**0.002**
DM	0.036 (-0.118- 0.189)	0.645	0.019 (-0.134- 0.173)	0.804
HCL	0.047 (-0.095- 0.190)	0.509	0.055 (-0.086- 0.195)	0.443
AF	0.045 (-0.088- 0.178)	0.501	0.034 (-0.101- 0.168)	0.622
CAD	0.023 (-0.121- 0.168)	0.751	0.155 (-0.002- 0.312)	0.053
Obesity (BMI > 30)	0.288 (0.133- 0.433)	**<0.001**	0.039 (-0.188- 0.110)	0.610
Chronic renal failure	0.157 (-0.027- 0.341)	0.094	0.140 (-0.042- 0.322)	0.130
Chronic hepatic failure	0.066 (-0.541- 0.673)	0.830	-0.072 (-0.432- 0.289)	0.695
Smoking	0.246 (0.058- 0.433)	**0.011**	0.094 (-0.058- 0.247)	0.225
Alcohol	0.218 (-0.001- 0.437)	0.051	-0.078 (-0.292- 0.135)	0.470
Malignancy	-0.336 (-0.548- -0.124)	**0.002**	-0.242 (-0.409- -0.076)	**0.005**
Depression	0.061 (-0.217- 0.339)	0.437	0.117 (-0.140- 0.375)	0.369
MCI/Dementia	-0.101 (-0.276- 0.074)	0.253	-0.197 (-0.435- 0.060)	0.132
Any kind of infections	0.278 (0.158- 0.339)	**<0.001**	0.347 (0.242- 0.452)	**<0.001**
**Baseline clinical variables/scales**				
NIHSS oa	0.011 (0.001- 0.022)	**0.037**	0.010 (0.001- 0.020)	**0.035**
Aphasia oa	0.014 (-0.118- 0.146)	0.834	0.168 (0.043- 0.293)	**0.009**
ICH-score oa	0.001 (-0.069- 0.072)	0.969	0.092 (0.045- 0.140)	**<0.001**
mRS before the ICH	-0.057 (-0.103- -0.010)	**0.018**	-0.028 (-0.078- 0.022)	0.267
**Etiology**				
Hypertension	0.150 (0.019- 0.281)	**0.025**	0.160 (0.046- 0.273)	**0.006**
CAA	-0.134 (-0.335- 0.067)	0.188	0.032 (-0.212- 0.277)	0.794
Mass	-0.344 (-0.588- -0.099)	**0.006**	-0.252 (-0.462- -0.041)	**0.019**
OAC	0.014 (-0.118- 0.146)	0.834	0.119 (-0.011- 0.249)	0.073
Vessel Pathology	NA	NA	-0.079 (-0.279- 0.120)	0.433
Other/Unknown	0.082 (-0.099- 0.262)	0.372	-0.058 (-0.205- 0.089)	0.437
**Location at baseline**				
DWM	0.146 (0.021- 0.271)	**0.023**	0.043 (-0.074- 0.161)	0.468
Lobar	-0.081 (-0.212- 0.051)	0.228	-0.075 (-0.195- 0.044)	0.214

Brainstem	0.324 (-0.103- 0.750)	0.135	0.147 (-0.076- 0.369)	0.175
Cerebellum	-0.199 (-0.397- -0.001)	**0.049**	-0.038 (-0.204- 0.128)	0.650
Left hemisphere	-0.052 (-0.181- 0.077)	0.427	0.078 (-0.040- 0.196)	0.192
Right hemisphere	0.048 (-0.081- 0.177)	0.463	-0.019 (-0.136- 0.097)	0.746
IVH oa	0.389 (-0.213- 0.990)	0.202	0.249 (-0.164- 0.662)	0.235
IVH extension	0.226 (0.107- 0.345)	**<0.001**	0.209 (0.086- 0.332)	**<0.001**
**ICH Volume [cm³]**	0.0004 (-0.004- 0.003)	0.802	0.001 (-0.002- 0.003)	0.546
**Invasive procedures**				
Surgical evacuation	-0.239 (-0.590- 0.112)	0.179	0.376 (0.208- 0.544)	**<0.001**
EVD	0.402 (0.218- 0.585)	**<0.001**	0.461 (0.269- 0.653)	**<0.001**

B; unstandardized correlation coefficient; OR, odds ratio; CI, confidence interval; ICH, intracerebral hemorrhage; PSD, post-stroke delirium; HP; hypertension; DM, Diabetes mellitus; HCL, Hypercholesterolemia; AF, atrial fibrillation; CAD, coronary artery disease; BMI, body mass index; MCI, mild cognitive impairment; NIHSS, National Institutes of Health Stroke Scale; oa, on admission; mRS, modified Rankin Scale; CAA, cerebral amyloid angiopathy; OAC, oral anticoagulants; DWM, deep white matter; IVH, intraventricular; EVD, external ventricular drain
Statistically significant values are given in bold

Table S3. Post-ICH without PSD patients' characteristics treated with melatonin vs. the control cohort after propensity matching.

Patients Characteristics	**Melatonin treated** n=62	**Control Cohort** n=87	**P-value**
**Demographics**			
Age, y median, (IQR)	70.00 (19.00)	72.00 (22.00)	0.764^
Sex n (%)			0.835*
Male	36 (58.1)	52 (59.8)	
Female	26 (41.9)	35 (40.2)	
**Risk Factors** n (%)			
HP	50 (80.6)	68 (78.2)	0.713*
DM	7 (11.4)	19 (21.8)	0.094*
HCL	15 (24.2)	18 (20.7)	0.612*
AF	13 (21.0)	24 (27.6)	0.357*
CAD	11 (17.7)	13 (15.1)	0.669*
Obesity (BMI > 30)	10 (16.1)	18 (20.7)	0.482*
Chronic renal failure	7 (11.3)	10 (11.5)	0.969*
Chronic hepatic failure	1 (1.6)	3 (3.4)	0.494*
Smoking	8 (12.9)	18 (20.7)	0.217*
Alcohol	3 (4.8)	9 (10.3)	0.223*
Malignancy	9 (14.5)	11 (12.6)	0.741*
Depression	4 (6.5)	4 (4.6)	0.621*
Cognition			0.783*#
Healthy	59 (95.2)	80 (94.1)	
MCI	3 (4.8)	5 (5.9)	
Dementia	0 (0.0)	0 (0.0)	
Any kind of infections	22 (35.5)	37 (42.5)	0.386*
**Baseline clinical variables/scales**			
NIHSS oa median (IQR)	3.00 (9.00)	5.00 (9.00)	**0.044^**
Aphasia oa n (%)	18 (29.0)	26 (29.9)	0.910*

ICH-score oa median (IQR)	1.00 (1.00)	1.00 (1.00)	**0.008^**
mRS before the ICH median (IQR)	0.0 (1.00)	0.00 (2.00)	**0.020^**
**Etiology**			
Hypertension	30 (48.3)	48 (55.2)	0.414*
CAA	5 (8.1)	4 (4.6)	0.381*
Mass	7 (11.3)	5 (5.7)	0.220*
OAC	18 (29.0)	22 (25.3)	0.611*
Vessel Pathology	4 (6.5)	10 (11.5)	0.298*
Other/Unknown	16 (25.8)	13 (14.9)	0.099*
**Location at baseline**			
DWM	22 (36.7)	43 (49.7)	0.126*
Lobar	27 (43.5)	30 (34.5)	0.262*
Brainstem	3 (4.8)	8 (9.2)	0.307*
Cerebellum	9 (14.5)	12 (14.0)	0.923*
Left hemisphere	36 (58.1)	52 (59.8)	0.835*
Right hemisphere	29 (46.8)	43 (49.4)	0.750*
IVH oa	0 (0.0)	3 (3.4)	0.140*
IVH extension	12 (19.4)	32 (36.8)	**0.022***
**ICH Volume [cm³]**	7.00 (13.00)	7.00 (20.00)	0.558^
**Invasive procedures** n (%)			
Surgical evacuation	7 (11.3)	11 (12.6)	0.803*
EVD	4 (6.5)	9 (10.3)	0.406*

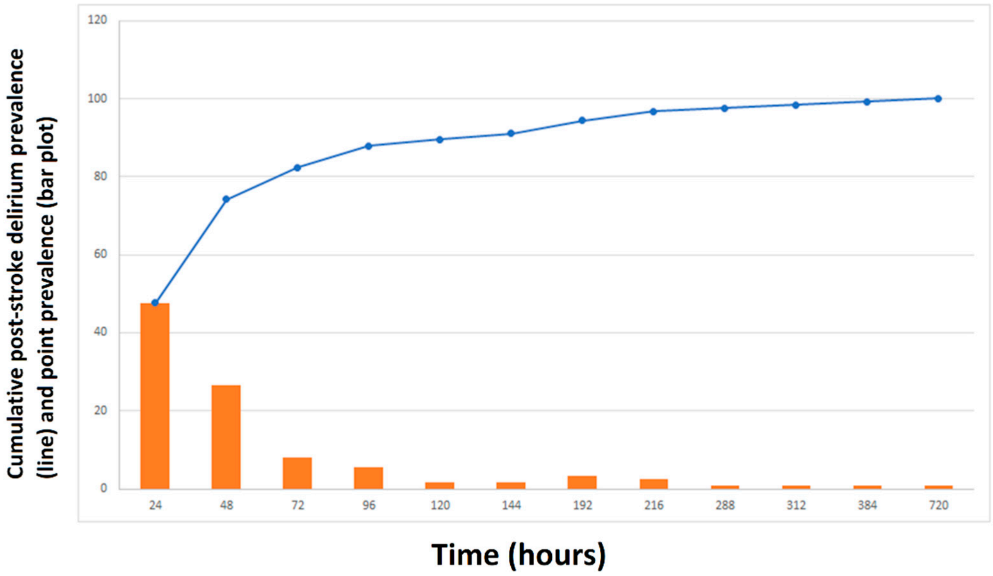

Figure S1. Cumulative post-stroke delirium prevalence (line) and point prevalence (bar plot).

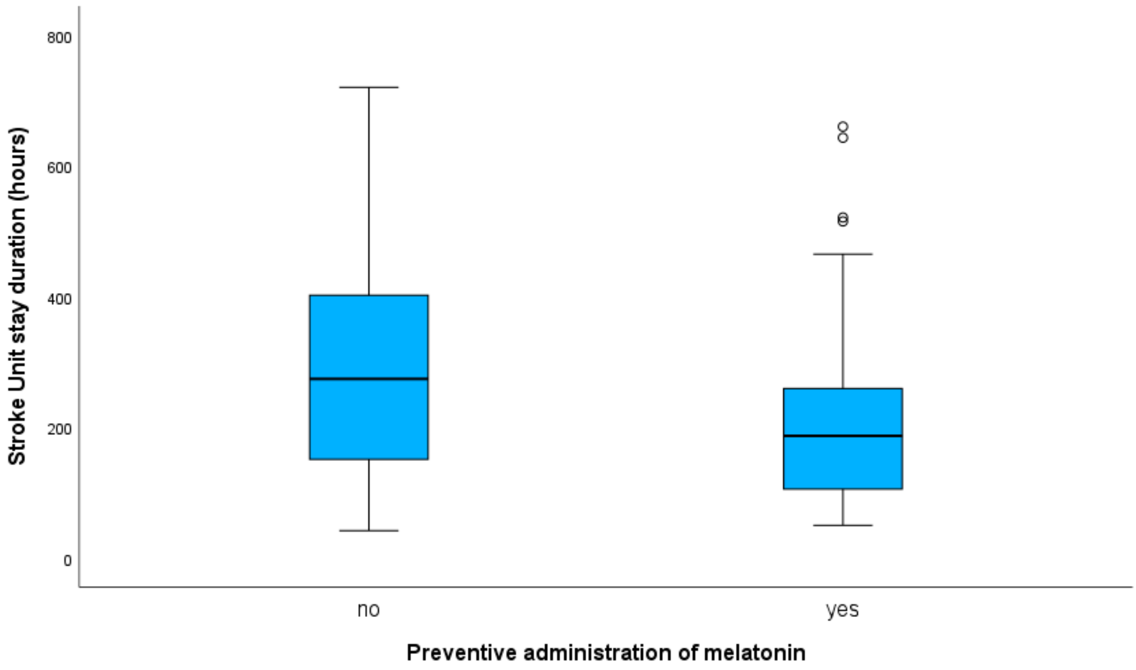

Figure S2. Box plots presenting data for stroke unit stay duration (hours) in respect to administration (or not) of melatonin in post-intracerebral hemorrhage patients with post-stroke delirium, who did not die during hospitalization. In the box plot, the black line within the box marks indicates the median, the boundary of the box closest to zero indicates the 1st quartile, and the boundary of the box farthest from zero indicates the 3rd quartile. Outliers with values more than 1.5 IQRs but less than 3 IQRs from the end of the box, denoted with a circle (o). Whiskers above and below the box indicate max and min values respectively, not including outliers. Median stroke unit stay duration of post-stroke delirium patients receiving melatonin was 187 hours, and 274 hours in the control group (p-value for Mann-Whitney U test=0.051).

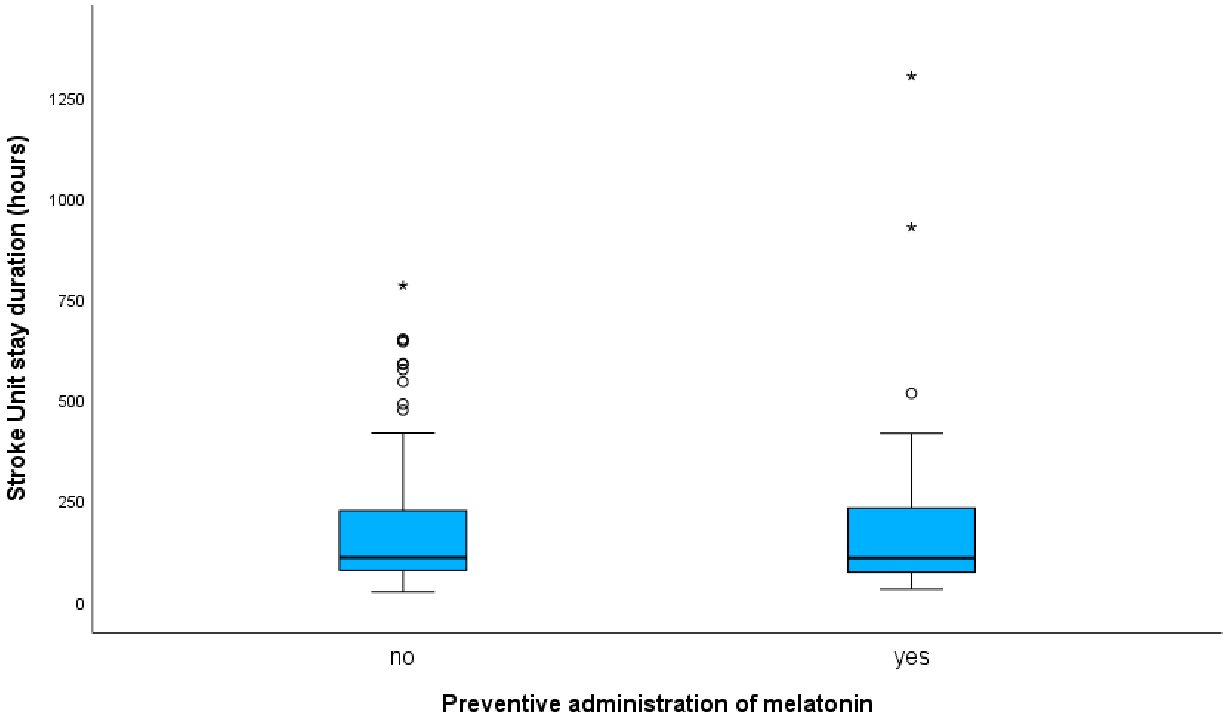

Figure S3. Box plots presenting data for stroke unit stay duration (hours) with respect to administration (or not) of melatonin in post-intracerebral hemorrhage patients without post-stroke delirium. In the box plot, the black line within the box marks indicates the median, the boundary of the box closest to zero indicates the 1st quartile, and the boundary of the box farthest from zero indicates the 3rd quartile. Outliers with values more than 3 interquartile ranges (IQRs) from the end of the box denoted with an asterisk (*). Outliers with values more than 1.5 IQRs but less than 3 IQRs from the end of the box, denoted with a circle (o). Whiskers above and below the box indicate max and min values respectively, not including outliers. Median stroke unit stay duration of patients without post-stroke delirium receiving melatonin was 108 hours, and 109 hours in the control group (p-value for Mann-Whitney U test=0.910).
